# *SREBF1c* and *SREBF2* gene polymorphisms are associated with acute coronary syndrome and blood lipid levels in Mexican population

**DOI:** 10.1371/journal.pone.0222017

**Published:** 2019-09-06

**Authors:** Gilberto Vargas-Alarcon, Hector Gonzalez-Pacheco, Oscar Perez-Mendez, Rosalinda Posadas-Sanchez, Guillermo Cardoso-Saldaña, Julian Ramirez-Bello, Galileo Escobedo, Betzabe Nieto-Lima, Jose Manuel Fragoso

**Affiliations:** 1 Department of Molecular Biology, Instituto Nacional de Cardiologia Ignacio Chavez, Mexico City, Mexico; 2 Atherosclerosis Study Group, Instituto Nacional de Cardiología Ignacio Chavez, Mexico City, Mexico; 3 Coronary Unit, Instituto Nacional de Cardiologia Ignacio Chavez, Mexico City, Mexico; 4 Department of Endocrinology, Instituto Nacional de Cardiologia Ignacio Chavez, Mexico City, Mexico; 5 Research Unit on Endocrine and Metabolic Diseases, Hospital Juarez de Mexico, Mexico City, Mexico; 6 Unit of the Experimental Medicine, Hospital General de Mexico, Dr. Eduardo Liceaga, Mexico City, Mexico; IMDEA Food Institute (CEI-UAM+CSIC), SPAIN

## Abstract

**Aim:**

It has recently been reported that the sterol regulatory element-binding transcription factors (SREBF-1c, and -2) contribute to the variation in the plasma lipids levels, which have an important role in the atherosclerotic plaque development. The aim of the present study was to evaluate whether the *SREBF1c* and *SREBF2* gene single nucleotide polymorphisms (SNPs) are associated with plasma lipids levels and ACS susceptibility in a case-control association study.

**Material and methods:**

A case-control study was carried out in 625 patients with ACS (82% men and 18% women, with a mean age of 57.97 ± 10.5 years) and 700 healthy controls (66% men and 34% women, with a mean age of 54.37 ± 7.65 years). The sample size was calculated for a statistical power of 80%. We genotyped three *SREBF1c* (rs2297508, rs11656665 and rs11868035) and three *SREBF2* (rs2267439, rs2267443, and rs2228314) gene polymorphisms by 5’ exonuclease TaqMan assays. The associations were evaluated by logistic regression under the co-dominant, dominant, recessive, over-dominant and additive inheritance models. The contribution of the genotypes on the plasma lipids levels was evaluated by Student’s t-test.

**Results:**

Under different models, the *SREBF1c* rs2297508 (OR = 1.50, *pC*_Res_ = 0.03), *SREBF1c* rs11656665 (OR = 1.35, *pC*_Dom_ = 0.02 and OR = 1.26, *pC*_Add_ = 0.02) and *SREBF2* rs2228314 (OR = 1.78, *pC*_Res_ = 0.03, OR = 1.27, *pC*_Add_ = 0.04) SNPs were associated with higher risk of ACS. On the other hand, the *SREBF1c* rs11868035 SNP was associated with lower risk of ACS (OR = 0.49, *pC*_Co-dom_ = 0.001, OR = 0.66, *pC*_Dom_ = 0.003, OR = 0.57, *P*_Res_ = 0.003 and OR = 0.71, *pC*_Add_ = 0.001). There was a statistically significant association of both *SREBF1c* rs11656665 and rs11868035 polymorphisms with plasma triglyceride levels.

**Conclusions:**

In summary, our data suggest the association of the *SREBF1c* and *SREBF2* SNPs with risk of developing ACS and with triglyceride levels in a Mexican population.

## Introduction

The acute coronary syndrome (ACS) is an important consequence of both atherosclerosis and abnormalities in lipid levels, constitutes a worldwide public health problem. Generally, the transition of a stable coronary atherosclerotic lesion into a ruptured or eroded plaque produces the clinical manifestations of the acute coronary syndrome [1,2].

Lipid variations are major risk factors of metabolic diseases, such as atherosclerosis, fatty liver disease, and diabetes [3]. In recent years, several studies have indicated that the sterol regulatory element-binding transcription factors (SREBF-1a, -1c, and -2) contribute to the variation of cholesterol, triglycerides, high-density lipoprotein-cholesterol (HDL-C), and low-density lipoprotein-cholesterol (LDL-C) levels [4–6]. In addition, these factors are partly responsible for the development of some diseases, such as type 2 diabetes mellitus (T2DM), schizophrenia, ischemia stroke, coronary artery disease, and hypercholesterolemia [7–12]. The SREBF-1a and SREBF-1c isoforms are both encoded in a single gene (sterol regulatory element binding transcription factor-1 (*SREBF-1)* located in chromosome 17p11.2). SREBF-1a stimulates the expression of both cholesterol and fatty acid biosynthesis genes, whereas SREBF-1c controls the expression of fatty acid, phospholipid and triglyceride biosynthetic genes [13–17].

On the other hand, SREBF-2 [encoded by the sterol regulatory element binding transcription factor-2 *(SREBF-2)* gene, located in chromosome 22q13.2] is preferentially involved in the regulation of cholesterol metabolism [14,15]. In this context, we postulated that *SREBF-1* and *SREBF-2* genes could be new candidates in the development of ACS due to their involvement in the regulation of the lipid biosynthesis; these molecules play an important role in the development of atherosclerotic plaque [1,2,5,6].

Recently three single nucleotide polymorphism (SNPs) of the SREBF-1c isoform: two in the 3’UTR region [positions A30225G (rs11868035) and G30009C (rs2297508)], and one in intron 1 [position IVS1 G954A (rs11656665)] have been associated with T2DM, hypercholesterolemia, and adiponectin levels [8,9,18]. Nonetheless, the association of these polymorphisms with other inflammatory diseases such as ischemic stroke and non-alcoholic fatty liver disease is controversial with negative results [11,19]. On the other hand, the SREBF-2 gene presents two relevant SNPs in the intronic region [at positions IVSI C8407T (rs2267439) and IVSI2 A1667G (rs2267443), respectively] and one in the exon 10 [at position G1784C Gly595Ala (rs2228314 has merged into rs4822063)]. These SREBF-2 variants have been associated with and increased risk for T2DM, hypercholesterolemia, premature coronary artery disease, and osteoarthritis [7,10,20,21].

Considering the prominent role of these genes in both lipid metabolism regulation and plasma lipid concentrations, the aim of this study was to establish the role of six polymorphisms (*SREBF-1c* 3’UTR *A3*0225*G*, *SREBF-1c* 3’UTR *G30009C*, *SREBF-1c IVS1 G954A*, *SREBF-2 IVSI C8407T*, *SREBF-2 IVSI2 A1667G*, and *SREBF-2 G1784C Gly595Ala)* in the susceptibility of developing ACS. Furthermore, we evaluated whether these polymorphisms were associated with plasma lipids levels in a Mexican population sample.

## Materials and methods

### Characteristics of the study population

The sample size calculation for unmatched cases and controls study, with a power of 80% and an alpha error of 0.05, showed that the total sample size needed to carry out this study was 528 Mexican mestizo individuals (264 patients with ACS and 264 control individuals) (http://www.openepi.com/SampleSize/SSCC.html). In this study, we included 1325 Mexican mestizo individuals (625 patients with ACS and 700 healthy controls unmatched by age or gender). From July 2007 to July 2017, we recruited 625 patients with ACS (82% men and 18% women, with a mean age of 57.97 ± 10.5 years) who were referred to the Instituto Nacional de Cardiologia Ignacio Chavez. From this patient population, 501 were diagnosed with myocardial infarction and 124 with unstable angina. ACS was diagnosed based on clinical characteristics, electrocardiographic changes and biochemical markers of cardiac necrosis (creatinine kinase isoenzymes, creatinine phosphokinase, or troponin I above the normal upper limit). The diagnosis of ACS was made according to guidelines from the European Society of Cardiology (ESC) and American College of Cardiology (ACC) [22,23]. The exclusion criteria were (1) patients with clear inflammatory pathologies on admission, such as infection established by clinical, laboratory or image investigations, and (2) patients with an autoimmune disease or cancer previously diagnosed or documented during their hospitalization. Moreover, we included 700 apparently healthy controls (66% men and 34% women, with a mean age of 54.37 ± 7.65 years) without a family history of premature coronary artery disease (pCAD) or atherosclerosis. The control group was recruited from June 2009 to June 2013 from blood bank donors and with the assistance of brochures posted in social service centers. These control subjects were nested in the cohort of the Genetics of Atherosclerosis Disease (GEA) Mexican study. The exclusion criteria included the use of anti-inflammatory drugs at the time of the study, congestive heart failure, and liver, renal, thyroid or oncological disease. In addition, the control subjects had a coronary calcium score of zero determined by computed tomography, indicating the absence of subclinical atherosclerosis in them [24]. All the included subjects were ethnically matched and considered Mexican mestizos only if they were at least third generation Mexicans born in the country.

### Ethics

The study complies with the Declaration of Helsinki. Ethical approval was provided by the Ethics and Research commission of Instituto Nacional de Cardiologia Ignacio Chavez—registration number: 17CI09012010. Written informed consent was obtained from all participants.

### Laboratory analyses

After a 12-h overnight fast, EDTA blood samples were drawn and centrifuged within 15 min after collection; plasma was separated into aliquots and immediately analyzed or frozen at −80°C until analysis. Cholesterol and triglycerides plasma concentrations were determined by enzymatic/colorimetric assays (Randox Laboratories, UK). The phosphotungstic acid-Mg^2+^ method was used to determine HDL-C concentrations. LDL-C was estimated in samples with a triglyceride level lower than 400 mg/dl, using the modified Friedewald formula [25]. Plasma lipid concentrations were determined within 24-h after blood sample collection. We followed the National Cholesterol Education Project (NCEP) Adult Treatment Panel (ATP III) guidelines and thus defined dyslipidemia with the following levels: cholesterol > 200 mg/dl, LDL-C > 130 mg/dl, HDL-C < 40 mg/dl, and triglyceride > 150 mg/dl. (http://www.nhlbi.nih.gov/guidelines/cholesterol/atp3_rpt.htm).

### Genetic analysis

DNA extraction was performed from peripheral blood in agreement with the method of Lahiri and Nurnberger [26]. The *SREBF-1c* 3’UTR *A30225G* (rs11868035), *SREBF-1c* 3’UTR *G30009C* (rs2297508), *SREBF-1c IVS1 G954A (rs11656665)*, *SREBF2 IVSI C8407T* (rs2267439), *SREBF2 IVSI2 A1667G* (rs2267443) and *SREBF2 G1784C Gly595Ala* (rs2228314) SNPs were genotyped using 5’ exonuclease TaqMan assays on a 7900HT Fast Real-Time PCR system according to manufacturer’s instructions (Applied Biosystems, foster City, USA). Samples previously sequenced for the different genotypes of the studied polymorphisms were included as positive controls.

### Functional prediction analysis

Two *in silico* programs [http://rulai.cshl.edu/cgi-bin/tools/ESE3/esefinder.cgi?process=home) and SNP Function Prediction (http://snpinfo.niehs.nih.gov/cgi-bin/snpinfo/snpfunc.cgi)] were used to predict the possible functional effect of the *SREBF1c* and *SREBF2* gene polymorphisms. Both programs (ESEfinder2.0 and SNPinfo) analyze the localization of the SNP (e.g. 5’-upstream, 3’-untranslated regions, intronic) and its possible functional effects such as amino acid changes in protein structure, transcription factor binding sites in promoter or intronic enhancer regions, and alternative splicing regulation by disrupting exonic splicing enhancers (*ESE*) or silencers [27,28].

### Statistical analysis

Continuous variables that are not normally distributed, such as age, body mass index (BMI), blood pressure, glucose, total cholesterol, HDL-C, LDL-C, were analyzed using the Mann-Whitney U test. Categorical variables, such as gender, hypertension, T2DM, and smoking habit were analyzed with the chi-squared test or Fisher’s exact test. Using co-dominant, dominant, recessive, over-dominant, and additive models, we analyzed the association of the polymorphisms with ACS by logistic regression. Multiple logistic models were built to identify the variables that best explained the risk of developing ACS. Models were constructed including one variable at the time, while final models included variables with biological relevance, statistical significance or both. Confounding bias was accepted when changes in estimated odds ratios (ORs) were equal to or greater than 10%. When a principal effect model was reached, effect modification was also tested and interaction terms were constructed between the polymorphisms and different variables; the terms were included in the model when the significance of the p-value was greater than or equal to 0.05. All p-values were corrected (pC) by the Bonferroni test. The values of pC < 0.05 were considered statistically significant, and all odds ratios (OR) are presented with 95% confidence intervals. The occurrence of the ACS in our population was based in the OR values: (a) OR = 1 does not affect the odds of developing ACS, (b) OR>1 is associated with higher odds of developing ACS, and (c) OR<1 is associated with lower odds of developing ACS. The linkage disequilibrium analysis (LD, D”) of the polymorphisms and the haplotypes construction was performed with Haploview version 4.1 (Broad Institute of Massachusetts Institute of Technology and Harvard University, Cambridge, MA, USA). We used the chi-squared test to evaluate Hardy-Weinberg equilibrium (HWE). Student’s t-test was used for establishing the contributions of the genotypes on the plasma lipids levels. Values are expressed as means ± SD, and statistical significance was set at p < 0.05. The analysis of data was performed with SPSS version 18.0 (SPSS, Chicago, Il) statistical package. The statistical power to detect an association with ACS was 0.80. We used the QUANTO software [http://biostats.usc.edu/software] to estimate the association.

## Results

### Characteristics of the study population

Demographic, clinical and biochemical characteristics of the ACS patients and healthy controls are shown in Table 1. There were significant differences between the ACS patients and healthy controls. Compared to healthy controls, the ACS patients had a higher frequency of T2DM, hypertension, dyslipidemia, and smoking habit. Conversely, the total cholesterol and LDL-C levels of ACS patients were lower than those of the control group; this effect may be due to their treatment with statins.

**Table 1:** Demographic characteristics and biochemical parameters of the study individuals.

		ACS (n = 625)		Healthy controls (n = 700)		*P* value
		Median	(percentile 25–75)	Median	(percentile 25–75)	
Age (years)		57	(51–65)	54	(49–59)	0.016
BMI (kg/m^2^)		27	(25–29)	28	(26–31)	0.214
Blood pressure (mmHg)	Systolic	130	(114–144)	115	(106–126)	<0.001
	Diastolic	80	(70–90)	72	(66–77)	<0.001
Glucose (mg/dl)		127	(102–188)	91	(84–99)	<0.001
Total cholesterol (mg/dl)		164	(128–198)	190	(164–210)	<0.001
HDL-C (mg/dl)		37	(32–44)	42	(35–53)	<0.001
LDL-C (mg/dl)		103	(76–133)	115	(94–134)	<0.001
Triglycerides (mg/dl)		149	(109–201)	151	(112–208)	0.218
Gender n (%)	Male	510	(82)	463	(66)	<0.001
	Female	115	(18)	237	(34)	
Smoking n (%)	Yes	225	(35)	155	(22)	<0.001
Hypertension n (%)	Yes	355	(57)	206	(29)	<0.001
Diabetes Mellitus n (%)	Yes	345	(55)	68	(10)	<0.001
Dyslipidemia n (%)	Yes	534	(85)	501	(71)	<0.001

Data are expressed as median and percentiles (25^th^-75^th^). *p-*values were estimated using Mann-Whitney U-test continuous variables and chi-square test for categorical values. ACS: Acute coronary syndrome patients.

### Allele and genotype frequencies

Genotype frequencies in the polymorphic sites were in HWE. The frequencies of the *SREBF2 IVSI C8407T* (rs2267439) and *SREBF2 IVSI2 A1667G* (rs2267443) polymorphisms were similar in ACS patients and healthy controls. Nonetheless, four SNPs [*SREBF-1c 3’UTR A30225G* (rs11868035), *SREBF-1c 3’UTR G30009C* (rs2297508), *SREBF-1c IVS1 G954A* (rs11656665), and *SREBF2 G1784C Gly595Ala* (rs2228314)] were associated with the presence of ACS (Table 2). Considering that the whole group of patients and controls were not age and sex matched, this analyses was adjusted by these variables. In order to corroborate the effect of these variables, we also performed a sub-analysis including a group of patients and controls age and sex matched. This sub-analysis showed similar results than those obtained with the whole group; the *SREBF-1c 3’UTR A30225G* (rs11868035), *SREBF-1c 3’UTR G30009C* (rs2297508), and *SREBF-1c IVS1 G954A* (rs11656665) SNPs were associated with the presence of ACS (S1 Table). Under a recessive model, the *G* allele of the *SREBF-1c* 3’UTR *G30009C* (rs2297508) SNP was associated with a higher risk of ACS (OR = 1.50, *pC*_Res_ = 0.03). In the same way, under dominant and additive models, the *A* allele of the *SREBF-1c IVS1 G954A* (rs11656665) SNP was associated with a higher risk of ACS (OR = 1.35, *pC*_Dom_ = 0.02 and OR = 1.26, *pC*_Add_ = 0.02, respectively). Moreover, under co-dominant, dominant, recessive and additive models, the *G* allele of the *SREBF-1c* 3’UTR *A30225G* (rs11868035) SNP was associated with a lower risk of ACS (OR = 0.49, *pC*_Co-dom_ = 0.001, OR = 0.66, *pC*_Dom_ = 0.003, OR = 0.57, *pC*_Res_ = 0.003 and OR = 0.71, *pC*_Add_ = 0.001). Finally, under recessive and additive models, the *G* allele of the *SREBF2 G1784C Gly595Ala* (rs2228314) SNP was associated with a higher risk of ACS (OR = 1.78, *pC*_Res_ = 0.03, OR = 1.27, *pC*_Add_ = 0.04, respectively). All models were adjusted for gender, age, blood pressure, BMI, glucose, total cholesterol, HDL-C, LDL-C, triglycerides, and smoking habit.

**Table 2:** Distribution of *SREBF-1c and SREBF-2* polymorphisms in ACS patients and healthy controls.

		Genotype frequency		MAF	Model	OR (95%CI)	*pC*
*SREBF-1c UTR’3*	*G30009C* (rs2297508)						
Control	*CC*	*CG*	*GG*				
(n = 698)	289 (0.414)	324 (0.464)	85 (0.121)	0.353	*Co-dominant*	1.53 (1.02–2.29)	0.10
					*Dominant*	1.14 (0.87–1.48)	0.34
ACS	240 (0.385)	281 (0.451)	102 (0.163)	0.390	*Recessive*	1.50 (1.03–2.18)	**0.03**
(n = 623)					*Over-dominant*	0.93 (0.72–1.20)	0.58
					*Log-additive*	1.18 (0.98–1.43)	0.08
*SREBF-1c IVS1*	*G954A* (rs11656665)						
Control	*GG*	*GA*	*AA*				
(n = 699)	318 (0.455)	298 (0.426)	83 (0.118)	0.331	*Co-dominant*	1.55 (1.03–2.31)	0.05
					*Dominant*	1.35 (1.04–3.76)	**0.02**
ACS	245 (0.395)	278 (0.448)	97 (0.156)	0.380	*Recessive*	1.35 (0.93–1.97)	0.11
(n = 620)					*Over-dominant*	1.16 (0.90–1.51)	0.25
					*Log-additive*	1.26 (1.04–1.52)	**0.02**
*SREBF-1c UTR’3*	*A30225G* (rs11868035)						
Control	*AA*	*AG*	*GG*				
(n = 695)	251 (0.361)	318 (0.457)	126 (0.181)	0.410	*Co-dominant*	0.49 (0.33–0.73)	0.001
					*Dominant*	0.66 (0.51–0.87)	0.003
ACS	268 (0.434)	269 (0.435)	80 (0.129)	0.347	*Recessive*	0.57 (0.40–0.83)	**0.003**
(n = 617)					*Over-dominant*	0.90 (0.69–1.17)	0.42
					*Log-additive*	0.71 (0.59–0.85)	**0.001**
*SREBF-2 IVS1*	*C8407T* (rs2267439)						
Control	*TT*	*TC*	*CC*				
(n = 692)	564 (0.815)	124 (0.179)	4 (0.006)	0.095	*Co-dominant*	2.98 (0.90–9.25)	0.14
					*Dominant*	1.17 (0.89–1.54)	0.26
ACS	489 (0.789)	120 (0.193)	10 (0.016)	0.113	*Recessive*	2.82 (0.88–9.05)	0.06
(n = 619)					*Over-dominant*	1.10 (0.83–1.46)	0.50
					*Log-additive*	1.21 (0.94–1.56)	0.14
*SREBF-2 IVS12*	*A1667* (rs2267443)						
Control	*GG*	*GA*	*AA*				
(n = 691)	465 (0.673)	197 (0.285)	29 (0.042)	0.184	*Co-dominant*	0.88 (0.50–1.56)	0.38
					*Dominant*	1.13 (0.90–1.42)	0.30
ACS	399 (0.645)	197 (0.318)	22 (0.035)	0.195	*Recessive*	0.84 (0.48–1.48)	0.55
(n = 618)					*Over-dominant*	1.17 (0.93–1.49)	0.18
					*Log-additive*	1.07 (0.88–1.30)	0.50
*SREBF-2*	*G1784C* (rs2228314)						
Control	*CC*	*CG*	*GG*				
(n = 689)	346 (0.502)	285 (0.413)	58 (0.084)	0.291	*Co-dominant*	1.87 (1.08–3.22)	0.08
					*Dominant*	1.24 (0.90–1.70)	0.19
ACS	284 (0.462)	252 (0.410)	78 (0.127)	0.332	*Recessive*	1.78 (1.05–3.00)	**0.03**
(n = 614)					*Over-dominant*	1.00 (0.72–1.38)	0.98
					*Log-additive*	1.27 (1.00–1.61)	**0.04**

ACS, Acute coronary syndrome; MAF, Minor allele frequency; OR, odds ratio; CI, confidence interval; pC, *p*-value. The p-values were calculated by the logistic regression analysis, and ORs were adjusted for gender, age, blood pressure, BMI, glucose, total cholesterol, HDL-C, LDL-C, triglycerides, and smoking habit.

### Linkage disequilibrium analysis

We analyzed haplotypes using the Haploview version 4.1 program. In this analysis, the *SREBF-1c* 3’UTR *A30225G* (rs11868035), 3’UTR *G30009C* (rs2297508), and *IVS1 G954A* (rs11656665) polymorphisms showed a moderate linkage disequilibrium (D’ > 0.60). Furthermore, eight haplotypes were constructed (Table 3); two (*AGG*, and *AGA*) of the eight haplotypes were associated with risk of developing ACS (OR = 1.93, 95% CI: 1.32–2.82, pC = 0.016, OR = 5.32, 95% CI: 3.08–9.20, pC = <0.008, respectively), whereas that the “*GGG*” haplotype was associated with a lower risk of developing ACS (OR = 0.50, 95% CI: 0.55–0.72, pC = 0.016). On the other hand, the analysis of the *SREBF2 IVSI C8407T* (rs2267439), *IVSI2 A1667G* (rs2267443), and *G1784C Gly595Ala* (rs2228314) SNPs showed a strong linkage disequilibrium (D’ > 0.90). The haplotype analysis showed five haplotypes with different distribution in ACS patients and healthy controls (Table 3). The “*TGG*” haplotype was associated with a higher risk of developing ACS (OR = 1.52, 95% CI: 1.06–2.17, pC = 0.02), while the “*TCG*” haplotype was associated with a lower risk of developing ACS (OR = 0.83, 95% CI: 0.70–0.98, pC = 0.03).

**Table 3:** Haplotype distribution of *SREBF1c* and *SREBF2* polymorphisms in ACS patients and healthy controls.

	ACS (n = 615)	Controls (n = 693)	OR	95%CI	*p*C
*SREBF1c* gene located in chromosome 17p11.2					
Haplotype	Hf	Hf			
*ACG*	0.470	0.501	0.88	0.75-1-02	0.11
*GGA*	0.232	0.241	0.95	0.79–1.13	0.61
*GCG*	0.049	0.063	0.77	0.55–1.09	0.17
*GGG*	0.036	0.070	0.50	0.55–0.72	**0.016**
*ACA*	0.063	0.044	1.47	1.04–2.08	0.26
*AGG*	0.061	0.032	1.93	1.32–2.82	**0.016**
*AGA*	0.059	0.012	5.32	3.08–9.20	**<0.008**
*GCA*	0.029	0.037	0.77	0.50–1.19	0.29
	ACS (n = 614)	Controls (n = 689)	OR	95%CI	*p*C
*SREBF2* gene located in chromosome 22q13.2					
Haplotype	Hf	Hf			
*TCG*	0.658	0.698	0.83	0.70–0.98	**0.03**
*TGA*	0.160	0.158	1.02	0.82–1.26	0.87
*CGG*	0.086	0.074	1.16	0.88–1.55	0.31
*TGG*	0.059	0.040	1.52	1.06–2.17	**0.02**
*CGA*	0.027	0.019	1.43	0.85–2.41	0.21

Abbreviations: Hf = Haplotype frequency, ACS = acute coronary syndrome, *pC =* corrected *p-value*. The order of the polymorphisms in the haplotypes is according to the positions in the chromosome. For the *SREBF1c* gene located in chromosome 17p11.2 [*SREBF-1c* 30225 *A/G* (rs11868035), *SREBF-1c* 30009 G/C (rs2297508), and *SREBF-1c IVS1-954 G/A (rs11656665)*], and for the *SREBF2* gene located in chromosome 22q13.2 [*SREBF2 IVSI+8407 C/T* (rs2267439), *SREBF2 1805 G/C* (rs2228314) and *SREBF2 IVSI2-1667 A/G* (rs2267443)]. The significant *p*-value is in bold.

### Association of polymorphisms with plasma lipids levels

We determined the contributions of these SNPs in plasma lipids levels, such as total cholesterol, HDL-C, LDL-C, triglycerides, and LDL-C / HDL-C ratio. For this analysis, we only selected the group of healthy individuals who fulfilled the above-mentioned inclusion criteria for control subjects. We did not include the analysis of lipids plasma levels in patients with ACS, because in the setting of the coronary syndrome, these levels may be altered by the use of the anti-dyslipidemic or anti-inflammatory drugs. Comorbidities, such as insulin resistance/T2DM, hypertension, and inflammatory processes can also mask the real impact of *SREBF1c* and *SREBF2* polymorphisms on plasma lipids. In this context, individual carriers of *AG/AA* (n = 226) genotypes of the *SREBF2 IVSI2 A1667G* (rs2267443) SNP had higher concentrations of cholesterol than *GG* homozygotes (194.62 ± 42.69 mg/dl, 189.06 ± 34.88 mg/dl, p < 0.034). On the other hand, the analysis of the *SREBF-1c 3’UTR A30225G* (rs11868035) SNP showed that individuals with *AG/GG* (n = 444) genotypes had lower triglyceride levels (170.68 ± 88.53 mg/dl, 183.75 ± 104.48 mg/dl, p < 0.041) than *AA* genotype carriers. In addition, carriers of the *GA/AA* (n = 381) genotypes of the *SREBF-1c IVS1 G954A* (rs11656665) SNP had a lower triglycerides concentration (169.61 ± 88.95 mg/dl, 181.70 ± 100.66 mg/dl, p < 0.046) than individuals with the *GG* genotype (Table 4). Additionally, we analyzed the association of the polymorphisms with cardiovascular risk factors, such as body mass index (BMI), blood pressure, glucose and their relation with *SRBEF-1c and SREBF2* SNPs. This analysis revealed that the subjects with *TC/CC* (n = 128) genotypes of the *IVSI C8407T* (rs2267439) SNP had a lower concentration of glucose (92.99 ± 20.38 mg/dl, 99.90 ± 33.90 mg/dl, p < 0.013), and a lower systolic blood pressure (115.17 ± 14.40 mmHg, 117.82 ± 16.35 mmHg, p = 0.046) than *TT* genotype.

**Table 4:** Association of the *SREBF1c* and *SREBF2* gene SNPs with plasma lipids levels and anthropometric characteristics in the healthy control group (*n = 700*).

*SREBF-1c*	*UTR’3 G30009C* (rs2297508)		p-value	*IVS1 G954A* (rs116656665)		p-value	*UTR’3 A30225G* (rs11868035)		p-value
	*CC + CG* *(n = 613)*	*GG* *(n = 85)*		*GA + AA* *(n = 381)*	*GG* *(n = 381)*		*GA + GG* *(n = 444)*	*AA* *(n = 251)*	
Parameters									
BMI (kg/m^2^)	28.4 ± 4.0	27.7 ± 4.0	0.069	28.3 ± 4.1	28.3 ± 4.0	0.495	28.1 ± 4.0	28.6 ± 4.2	0.096
Blood pressure (mmHg)									
Systolic	117 ± 16	116 ± 15	0.214	117 ± 16	118 ± 16	0.147	117 ± 18	119 ± 15	0.284
Diastolic	73 ± 9	71 ± 8	0.117	72 ± 9	72 ± 8	0.464	73 ± 9	72 ± 9	0.276
Glucose (mg/dl)	99 ± 32	95 ± 28	0.129	97 ± 29	100 ± 35	0.161	98 ± 30	100 ± 36	0.158
Total cholesterol (mg/dl)	191 ± 38	188 ± 38	0.239	199 ± 38	191 ± 37	0.454	191 ± 38	191 ± 38	0.467
HDL-C (mg/dl)	45 ± 13	43 ± 13	0.198	43 ± 13	44 ± 14	0.397	45 ± 13	44 ± 14	0.432
LDL-C (mg/dl)	116 ± 31	115 ± 34	0.406	117 ± 32	115 ± 32	0.211	116 ± 30	115 ± 34	0.341
Triglycerides (mg/dl)	174 ± 94	179 ± 98	0.340	170 ± 89	182 ± 100	**0.043**	171 ± 88	184 ± 104	**0.041**
Ratio LDL-C/HDL-C	2.80 ± 1.08	2.85 ± 1.05	0.350	2.82 ± 1.11	2.78 ± 1.02	0.324	2.80 ± 1.05	2.79 ± 1.12	0.365
*SREBF2*	*IVS1 C8407T* (rs2267439)		p-value	*IVS12 A1667G* (rs2267443)		p-value	*G1784C* (rs2228314)		p-value
	*TC + CC* *(n = 128)*	*TT* *(n = 564)*		*GA + AA* *(n = 226)*	*GG* *(n = 465)*		*CG + GG* *(n = 631)*	*GG* *(n = 58)*	
Parameters									
BMI (kg/m^2^)	28.4 ± 4.3	28.3 ± 4.0	0.392	28.1 ± 4.1	28.4 ± 4.0	0.225	28.4 ± 4.2	27.8 ± 4	0.145
Blood pressure (mmHg)									
Systolic	115 ± 14	118 ± 16	**0.043**	117 ± 16	117 ± 16	0.435	117 ± 16	116 ± 16	0.322
Diastolic	71.9 ± 8	72.6 ± 9	0.200	72 ± 9	72 ± 9	0.312	72 ± 9	72 ± 8	0.388
Glucose (mg/dl)	93 ± 20	100 ± 34	**0.013**	100 ± 35	98 ± 30	0.200	98.4 ± 31.4	101 ± 38	0.275
Total cholesterol (mg/dl)	191 ± 30	191 ± 39	0.488	194 ± 43	189 ± 35	**0.034**	191 ± 37	192 ± 45	0.390
HDL-C (mg/dl)	43 ± 13	44 ± 13	0.433	46 ± 14	44 ± 13	0.079	44 ± 13	46 ± 15.4	0.232
LDL-C (mg/dl)	116 ± 28	116 ± 33	0.464	117 ± 35	115 ± 30	0.149	116 ± 32	112 ± 28	0.191
Triglycerides (mg/dl)	183 ± 104	174 ± 93	0.167	175 ± 98	176 ± 93	0.482	176 ± 96	166 ± 82	0.214
Ratio LDL-C/HDL-C	2.81 ± 0.95	2.81 ± 1.10	0.476	2.79 ± 1.05	2.82 ± 1.09	0.312	2.82 ± 1.09	2.72 ± 1.01	0.232

Abbreviations: BMI = Body mass index, HDL = High-density lipoprotein–cholesterol, LDL: Low density lipoprotein, *p* = *p-value*. Data of BMI, blood pressure, glucose, total cholesterol, HDL-C, LDL-C, triglycerides and Ratio LDL-C/HDL-C are expressed as mean ± SD adjusted by gender and age.

### Functional prediction

The functional prediction analysis showed that the presence of the *G* allele of the *SREBF1c 3’UTR G30009C* (rs2297508) polymorphism potentially produces a binding motif for Srp55 protein. The analysis also revealed that the *G* allele of the *SREBF2* G*1784C Gly595Ala* (rs2228314) polymorphism may generate binding motifs for SF2/ASF protein. In contrast, the analysis of the *SREBF1c* 3’UTR *G30009C* (rs2297508), *SREBF1c IVS1 G954A* (rs11656665), *SREBF2 IVSI C8407T* (rs2267439), and *SREBF2 IVSI2 A1667G* (rs2267443) polymorphisms did not exhibit evidence of potentially functional motifs.

## Discussion

In our study, a similar distribution of *IVSI C8407T* (rs2267439) and *IVSI2 A1667G* (rs2267443) SNPs of the *SREBF2* gene were observed in ACS patients and healthy controls. Nonetheless, we also found that the presence of the *G* allele of *SREBF2* G*1784C Gly595Ala* (rs2228314) SNP was associated with a risk of developing ACS. Contrary to our findings, Robinet et al. reported that the *C* allele of the *G1784C* (rs2228314) SNP is associated with development of intima-media thickness and early-stage carotid atherosclerosis in a French population [17]. In same way, Miserez et al. reported that the *C* allele of *G1784C* (rs2228314) SNP contributes to the development of hypercholesterolemia and increased plasma cholesterol levels in Swiss and Israeli populations [7]. In contrast to these data, studies in Asian populations report a lack of association of the G*1784C Gly595Ala* (rs2228314) SNP with premature coronary artery disease and coronary heart disease [10,29]. Additionally, the haplotype analysis showed that the “*TGG”* haplotype, composed of *IVSI C8407T* (rs2267439), G*1784C Gly595Ala* (rs2228314), and *IVSI2 A1667G* (rs2267443) SNPs, was associated with the risk of developing ACS. In contrast, the “*TCG”* haplotype showed a lower frequency in ACS patients. As can be seen, the “*TGG*” haplotype is differentiated by the presence of the *G* allele of the *G1784C Gly595Ala* (rs2228314) polymorphism; the *G* allele marks the risk haplotype, while the *C* allele characterizes the protective haplotype. Of note, in the SNP independent analysis, we found an association between the *G* allele and the presence of ACS; this allele has thus an important role in the development of the disease.

On the other hand, we found an association of the *A* allele of the *SREBF-1c* gene *IVS1 G954A* (rs11656665) SNP with a higher risk of developing ACS in the study population. A meta-analysis showed that the *A* allele of *SREBF-1c IVS1 G954A* (rs11656665) SNP is associated with lower adiponectin levels [18]. This meta-analysis is in accord with our findings since hypoadiponectinemia may contribute to a higher cardiovascular risk. In addition, our results demonstrated that the 3’UTR *A30225G* (rs11868035) and 3’UTR *G30009C* (rs2297508) SNPs of the *SREBF-1c* gene play an important role in the presence of ACS. To the best of our knowledge, this study is the first to describe the association between these polymorphisms with the presence of ACS. In this context, the association of these SNPs with several inflammatory diseases in different populations is controversial. In agreement with our findings, Felder et al. reported that *G* allele of the *SREBF-1c* 3’UTR *G30009C* SNP is associated with a risk of developing T2DM in a French population [9]. Similarly, in a Danish population, Grarup et al. reported in a meta-analysis study that the *G* allele is associated with a higher risk of T2DM [30]. However, Peng et al. studied a Chinese Han population and reported that the 3’UTR *A*30225*G* and 3’UTR G30009C polymorphisms were not associated with risk of non-alcoholic fatty liver disease (NAFLD) [19]. With regard to the 3’UTR *A30225G* (rs11868035) polymorphism, we found that the *G* allele was associated with lower risk of developing ACS. In contrast, Liu et al. reported that *G* allele of the 3’UTR *A30225G* (rs11868035) SNP in a Chinese population increased the risk of developing T2DM (OR = 1.76) [31]. In line with our findings, studies in Caucasian populations have shown that the *A* allele of the *SREBF-1c* 3’UTR *A30225G* (rs11868035) SNP is associated with a higher risk of developing T2DM [30,32,33]. In addition, we found that the *AGG* and *AGA* haplotypes were associated with increased risk of developing ACS. However, as far as we know, this study is first that describes haplotypes between these polymorphisms.

Moreover, in our study, the association of the *SREBF2 G1784C* (rs2228314) and *SREBF-1c* 3’UTR *A30225G* (rs11868035) polymorphisms with the presence ACS were positive, but controversial with other populations. We think that the association of the *SREBF1c* and *SREBF2* polymorphisms could be due to the classical cardiovascular risk factors, and the environmental factors such as diet, exercise and lifestyle that have an important role in the development of the inflammatory diseases [19,33], as well as, to the fact that the allelic distribution of these polymorphisms varies according to the ethnic origin of the study populations. In this context, data obtained from the National Center for Biotechnology Information, showed that Caucasian population presents a higher frequency of the *G* allele of the 3’UTR *A30225G* (rs11868035) SNP (72%) when compared to Mexican Mestizos and Asian population that present a lower frequency of the *G* allele (41% and 16%, respectively). Concerning the *G1784C* (rs2228314) SNP the Mexican Mestizos presents a lower frequency of the G allele (29%) when compared to Asian and Caucasian populations (80% and 77%, respectively) (https://www.ncbi.nlm.nih.gov/variation/tools/1000genomes/). Owing to the specific genetic characteristics of the Mexican population, we consider that additional studies are needed in a larger number of individuals and in other populations with different ethnicities. This research could help define the true role of *SREBF1c and SREBF2* polymorphisms as risk or protective markers in the development of ACS and others cardiovascular events.

We further determined the effect of the *SREBF2* and *SREBF-1c* gene polymorphisms on plasma lipid levels using genotype groups. In this context, the analysis of the *G1784C Gly595Ala* (rs2228314) SNP did not show changes in the plasma lipid levels. The *G* and *T* alleles of the *IVSI2 A1667G* (rs2267443) and *IVS1 C8407T* (rs2267439) SNPs were not associated with risk of developing ACS. Nonetheless, we found an association of these two SNPs with not only higher cholesterol and glucose plasma concentrations, but also higher systolic blood pressure (p < 0.05). In contrast to our data, Galavi et al. reported that the *IVSI2 A1667G* (rs2267443) and *IVS1 C8407T* (rs2267439) SNPs were not associated with plasma lipids levels in T2DM patients [21]. On the other hand, Grarup et al. reported that the *G* allele of the 3’UTR *G30009C* (rs2297508) SNP was associated with higher cholesterol and glucose plasma levels. In addition, the authors showed that the *A* allele of 3’UTR *A30225G* (rs11868035) was associated with higher cholesterol plasma levels [30]. By the same token, Musso et al. reported that the *A* allele of *SREBF-1c* 3’UTR *A30225G* (rs11868035) was associated with higher triglyceride and LDL-C plasma levels [33]. However, in this study population, we found that the *SREBF-1c* 3’UTR *G30009C* (rs2297508) SNP was not related with a change of plasma lipid levels. In addition, we found that the *G* allele of the *SREBF-1c* 3’UTR *A30225G* (rs11868035) was associated with lower triglyceride levels. In the same way, Peng et al. reported that the *G* allele of the *SREBF-1c* 3’UTR *A30225G* (rs11868035) was associated with lower triglyceride levels in non-alcoholic fatty liver disease [19]. Additionally, using bioinformatics tools we determined the potential effect of the *SREBF2* and *SREBF-1c* gene polymorphisms associated with ACS. The analysis of the *SREBF1c* 3’UTR *G30009C* (rs2297508), *SREBF1c IVS1 G954A* (rs11656665), *SREBF2 IVSI C8407T* (rs2267439), and *SREBF2 IVSI2 A1667G* (rs2267443) polymorphisms did not exhibit evidence of potential functional motifs. Nonetheless, the analysis of the *SREBF2 G1784C Gly595Ala* (rs2228314) SNP showed that the *G* allele generates a binding site for the SRp55 protein. In the same way, the analysis of the *SREBF1c* 3’UTR *G30009C* (rs2297508) polymorphism showed that the *G* allele generates a binding site for the SF2/ASF proteins. These proteins have multiple functions in the pre-mRNA splicing process, as well as in the regulation of alternative splicing [34,35]. In our opinion, the specific correlation of these polymorphisms with plasma lipid levels deserves to be addressed in future studies.

### Limitations

Unfortunately, in our study was not possible to match patients and controls by age and gender, considering this, the analysis was adjusted by these variables. On the other hand, a sub-analysis in a groups age and gender matched was done. In this case, the results were similar to those reported in the whole group of studied individual. Moreover, our results in Mexicans should be reproduced in other ethnic groups to have a better definition about the contribution of the *SREBF1c* and *SREBF2* gene polymorphisms on the lipid profile as well as their role as possible markers of risk or protection against the development of ACS.

In summary, this study demonstrated that, in the Mexican population, three polymorphisms were associated with a high risk of developing ACS: the *SREBF-1c* 3’UTR *G30009C* (rs2297508) and *SREBF-1c IVS1 G954A* (rs11656665) polymorphisms of the *SREBF1c* gene, and *SREBF2 G1784C Gly595Ala* (rs2228314) of the *SREBF2* gene. On the other hand, the *SREBF-1c* 3’UTR *A30225G* (rs11868035) polymorphism of the *SREBF1c* gene was associated with a lower risk of developing ACS. There was also a statistically significant association of both *SREBF-1c IVS1 G954A* (rs11656665) and *SREBF-1c* 3’UTR *A30225G* (rs11868035) polymorphisms with lower triglyceride levels.

## Supporting information

S1 Table Sub-analysis of the *SREBF2* and *SREBF-1c* gene polymorphisms. (DOC)

## Abbreviations

*ACS*: Acute coronary syndrome
*HDL-C*: High-density lipoprotein–cholesterol
*LDL*: Low-density lipoprotein–cholesterol
*SNP*: Single nucleotide polymorphism
*SREBF1c*: Sterol regulatory element binding transcription factor 1c
*SREBF2*: Sterol regulatory element binding transcription factor 2
*T2DM*: Type 2 diabetes mellitus
