# Supplementary material for: SREBF1c and SREBF2 gene polymorphisms are associated with acute coronary syndrome and blood lipid levels in Mexican population
Source: PLoS One. 2019 Sep 6;14(9):e0222017. doi: 10.1371/journal.pone.0222017 (PMC6730878; doi:10.1371/journal.pone.0222017)
Supplement: S1 Table — (DOC) [file pone.0222017.s001.doc]

S1 Table. Distribution of *SREBF-1c and SREBF-2* polymorphisms in ACS patients and healthy controls

|  |  | Genotype frequency |  | MAF | Model | OR (95%CI) | *pC* |
| --- | --- | --- | --- | --- | --- | --- | --- |
| *SREBF-1c UTR’3* | *G30009C* (rs2297508) |  |  |  |  |  |  |
| Control | *CC* | *CG* | *GG* |  |  |  |  |
| (n=575) | 238 (0.413) | 258 (0.448) | 79 (0.137) | 0.361 | *Co-dominant* | 1.53 (0.99-2.37) | 0.09 |
|  |  |  |  |  | *Dominant* | 1.09 (0.81-1.47) | 0.55 |
| ACS | 226 (0.393) | 254 (0.441) | 95 (0.166) | 0.390 | *Recessive* | 1.55 (1.04-2.32) | **0.033** |
| (n=575) |  |  |  |  | *Over-dominant* | 0.87 (0.65-1.16) | 0.58 |
|  |  |  |  |  | *Log-additive* | 1.1¡7 (0.95-1.44) | 0.08 |
| *SREBF-1c IVS1* | *G954A* (rs11656665) |  |  |  |  |  |  |
| Control | *GG* | *GA* | *AA* |  |  |  |  |
| (n=575) | 258 (0.448) | 250 (0.434) | 67 (0.116) | 0.333 | *Co-dominant* | 1.71 (1.10-2.67) | 0.06 |
|  |  |  |  |  | *Dominant* | 1.29 (0.96-1.74) | 0.09 |
| ACS | 230 (0.400) | 254 (0.441) | 91 (0.158) | 0.379 | *Recessive* | 1.57 (1.04-2.38) | **0.03** |
| (n=575) |  |  |  |  | *Over-dominant* | 1.03 (0.77-1.38) | 0.84 |
|  |  |  |  |  | *Log-additive* | 1.27 (1.03-1.57) | **0.022** |
| *SREBF-1c UTR’3* | *A30225G* (rs11868035) |  |  |  |  |  |  |
| Control | *AA* | *AG* | *GG* |  |  |  |  |
| (n=575) | 219 (0.365) | 252 (0.438) | 113 (0.196) | 0.415 | *Co-dominant* | 0.58 (0.38-0.90) | 0.049 |
|  |  |  |  |  | *Dominant* | 0.76 (0.56-1.02) | 0.072 |
| ACS | 250 (0.434) | 253 (0.440) | 72 (0.125) | 0.345 | *Recessive* | 0.64 (0.43-0.95) | **0.027** |
| (n=575) |  |  |  |  | *Over-dominant* | 0.98 (0.73-1.31) | 0.89 |
|  |  |  |  |  | *Log-additive* | 0.78 (0.63-0.96) | **0.017** |
| *SREBF-2 IVS1* | *C8407T* (rs2267439) |  |  |  |  |  |  |
| Control | *TT* | *TC* | *CC* |  |  |  |  |
| (n=575) | 460 (0.800) | 111 (0.193) | 4 (0.007) | 0.103 | *Co-dominant* | 3.99 (0.99-16.13) | 0.13 |
|  |  |  |  |  | *Dominant* | 1.18 (0.82-1.71) | 0.37 |
| ACS | 457 (0.794) | 110 (0.191) | 8 (0.014) | 0.096 | *Recessive* | 1.92 (0.97-15.82) | 0.10 |
| (n=575) |  |  |  |  | *Over-dominant* | 1.08 (0.74-1.57) | 0.71 |
|  |  |  |  |  | *Log-additive* | 1.25 (0.89-1.75) | 0.19 |
| *SREBF-2 IVS12* | *A1667* (rs2267443) |  |  |  |  |  |  |
| Control | *GG* | *GA* | *AA* |  |  |  |  |
| (n=575) | 390 (0.678) | 156 (0.271) | 29 (0.050) | 0.186 | *Co-dominant* | 0.56 (0.26-1.16) | 0.18 |
|  |  |  |  |  | *Dominant* | 1.03 (0.75-1.41) | 0.83 |
| ACS | 375 (0.648) | 180 (0.313) | 22 (0.038) | 0.194 | *Recessive* | 0.53 (0.26-1.11) | 0.10 |
| (n=575) |  |  |  |  | *Over-dominant* | 1.17 (0.85-1.61) | 0.33 |
|  |  |  |  |  | *Log-additive* | 0.95 (0.73-1.23) | 0.68 |
| *SREBF-2* | *G1784C* (rs2228314) |  |  |  |  |  |  |
| Control | *CC* | *CG* | *GG* |  |  |  |  |
| (n=575) | 283 (0.492) | 238 (0.413) | 54 (0.094) | 0.300 | *Co-dominant* | 1.33 (0.80-2.21) | 0.54 |
|  |  |  |  |  | *Dominant* | 1.11 (0.83-1.49) | 0.47 |
| ACS | 270 (0.469) | 235 (0.408) | 70 (0.121) | 0.326 | *Recessive* | 1.29 (0.80-2.11) | 0.29 |
| (n=575) |  |  |  |  | *Over-dominant* | 1.01 (0.75-1.36) | 0.93 |
|  |  |  |  |  | *Log-additive* | 1.12 (0.90-1.40) | 0.31 |

ACS, Acute coronary syndrome; MAF, Minor allele frequency; OR, odds ratio; CI, confidence interval; pC, *p*-value. The p-values were calculated by the logistic regression analysis, and ORs were adjusted for blood pressure, BMI, glucose, total cholesterol, HDL-C, LDL-C, triglycerides, and smoking habit.
